# Circulating MiR-133a as a Biomarker Predicts Cardiac Hypertrophy in Chronic Hemodialysis Patients

**DOI:** 10.1371/journal.pone.0103079

**Published:** 2014-10-14

**Authors:** Ping Wen, Dan Song, Hong Ye, Xiaochun Wu, Lei Jiang, Bing Tang, Yang Zhou, Li Fang, Hongdi Cao, Weichun He, Yafang Yang, Chunsun Dai, Junwei Yang

**Affiliations:** 1 Center for Kidney Disease, Second Affiliated Hospital, Nanjing Medical University, Nanjing, China; 2 Department of Radiology, Second Affiliated Hospital, Nanjing Medical University, Nanjing, China; 3 Department of Nephrology, Affiliated Wuxi Hospital, Nanjing Medical University, Wuxi, China; Loyola University Chicago, United States of America

## Abstract

**Background:**

MicroRNAs (miRNAs) are small ribonucleotides regulating gene expression. MicroRNAs are present in the blood in a remarkably stable form and have emerged as potential diagnostic markers in patients with cardiovascular disease. Our study aimed to assess circulating miR-133a levels in MHD patients and the relation of miR-133a to cardiac hypertrophy.

**Methods:**

We profiled miRNAs using RNA isolated from the plasma of participants. The results were validated in 64 MHD patients and 18 healthy controls.

**Results:**

Levels of plasma miR-133a decreased in MHD patients with LVH compared with those in healthy controls. Plasma miR-133a concentrations were negatively correlated with LVMI and IVS. After single hemodialytic treatment, plasma miR-133a levels remained unchanged. Cardiac Troponin I and T were not associated with LVMI and IVS.

**Conclusions:**

Our observations supplied the possibility that circulating miR-133a could be a surrogate biomarker of cardiac hypertrophy in MHD patients.

## Introduction

Cardiovascular disease (CVD) is the main complication in patients with chronic kidney disease (CKD) and end stage renal disease (ESRD). More importantly, CVD is the leading cause of death in ESRD patients, accounting for 50% of all deaths in renal replacement therapy patients. In ESRD patients, a major pathophysiologic process frequently occurring in uremic hearts is left ventricular hypertrophy (LVH). Even though coronary artery disease and arrhythmia are not uncommon, LVH is the most frequent cardiovascular manifestation in these patients [1], [2]. In addition, LVH is a very strong independent predictor of cardiovascular mortality not only among patients with hypertension but also among ESRD patients [3], [4].

To date, several imaging modalities such as echocardiography (ECHO), magnetic resonance imaging (MRI) and computerized tomography have been performed to measure LVH [5], [6], [7]. ECHO is widely available, noninvasive, and has been demonstrated to be of reasonable accuracy in the assessment of LVH. However, ECHO might not be able to detect LVH in all dialysis patients [8]. In addition, there are studies implied serum biomarkers like arterial vasopressin, aldosterone and cardiac Troponin T appear to predict LVH [9], [10]. However, stable and accurate serum biomarkers are not found, the present study evaluates whether a new biomarker for LVH can improve detection of LVH.

Recent studies have demonstrated that microRNAs (miRNAs) are present in the human circulation in a cell-free form and can be detected in circulating blood, thus may serve as a new class of blood-based biomarkers [11]. Numerous studies reported altered plasma or serum levels of various miRNAs in patients with cardiovascular diseases, including acute myocardial infarction [12], [13], myocarditis [14], acute and chronic heart failure [14], [15] and stable coronary artery disease [16]. Previous studies have found that miR-133a level was decreased in hypertrophic heart [17]. In study presented here, we measured plasma miR-133a in MHD patients and healthy controls and analyzed the relationship between miR-133a level and cardiac hypertrophy.

## Subjects and Methods

### Ethical statement

All of the following details of the study were approval by the responsible ethics committee of Nanjing Medical University (Permit Number: KY2013019). And the written informed consent was supplied by the patients before the study.

### Patients

The study population consists of 64 patients with ESRD undergoing regular hemodialysis from one clinical center and 18 healthy controls from one health examination center. Two blood samples of each patient were collected: one before hemodialysis another after hemodialysis, while one blood sample was obtained from each control. All the patients were evaluated with transthoracic echocardiography and 8 of them were measured with cardiac magnetic resonance.

### Left ventricular mass index estimated by echocardiography

We measured the following parameters on the M-mode echocardiogram: left ventricular diastolic dimension (LVDd, cm), interventricular septum thickness (IVS, cm), and left ventricular posterior wall thickness (LVPW, cm). LV mass was calculated according to Devereux's formula [18]: left ventricular mass (g) = 1.04×[(LVDd+IVS+LVPW)^3^−(LVDd)]^3^−13.6, where 1.04 (g/cm^3^) is the specific gravity of the myocardium. It is the measurements of the peak value of R wave on the electrocardiograph. The left ventricular mass index (LVMI, g/m^2^) was defined as left ventricular mass divided by body surface area (m^2^).

### Biochemical analyses

Plasma cardiac Troponin I (CA: DRE11443) and Troponin T (CA: DRE11403) were measured using the ELISA kit (fengxiang biotechnology company, Shanghai, China) by sandwich ELISA. The detection range were 10∼300 ng/L and 5∼220 ng/L respectively.

### Plasma microRNA determination

Total RNA was extracted from plasma as previously described [19]. Quantitative RT-PCR was carried out using TaqMan miRNA probes (Applied Biosystems; Foster City, CA) according to the manufacturer's instructions. Real-time PCR was performed using a TaqMan PCR kit on an Applied Biosystems 7300 Sequence Detection System. The primer sequences of miR-133a was UUGGUCCCCUUCAACCAGCUGU. The mixtures of let-7d, let-7g and let-7i were used for our reference gene. To evaluate the change of miR-133a levels before and after hemodialysis, Δct was used, which can be calculated as the following equation: Δct = ct value (miR-133a)- ct value (let-7dgi).

### Statistical analyses

Data are presented as mean±SE unless otherwise described. To test the differences of miR-133a between MHD patients and healthy controls, t-test was used. Correlations were assessed using liner regression analysis and univariate logistic regression analysis method. Paired t-test was used to evaluate the change of miR-133a levels, Troponin I/T levels after hemodialysis. PASW statistics, version 18 was used to perform statistical analyses. A probability value <0.05 was considered to indicate statistical significance.

## Results

### Patient characteristics

A total of 82 subjects were enrolled in this study and 64 of them were ESRD patients receiving maintenance hemodialysis treatment. Clinical characteristics of all patients are shown in Table 1. 18 volunteers without documented kidney or heart diseases were used as healthy controls. Among the 64 patients, 40 had left ventricular hypertrophy which is defined as LVMI>125 g/m^2^ in male and LVMI >115 g/m^2^ in female. Thereby patients were divided into two groups according to the LVMI value. The traditional risk factors of LVH such as age, hypertension, and anemia were not significantly different between patients with LVH and without LVH. Among patients with LVH, there were 6 patients have diabetes. In contrast, all of the patients without LVH did not suffer the diabetes, suggesting that diabetes is a strong risk factor of LVH. There were no significant differences of laboratory indexes and medications between the two groups.

**Table 1:** Clinical characteristics of the study population.

characteristics	controls	MHD without LVH	MHD with LVH
Number	18	24	40
Men, n(%)	12(66.7)	14(58.3)	26(65)
Median age(range) (yr)	42(27–59)	46(29–69)	54(44–85)
Cardiovascular history, n(%)			
Myocardial infarction	0(0)	1(4.2)	0(0)
PTCA	0(0)	1(4.2)	0(0)
CABG	0(0)	0(0)	0(0)
Pacemaker	0(0)	0(0)	1(4.2)
Hypertension	0(0)	22(91.7)	35(87.5)
Diabetes	0(0)	0(0)	6(15)
Hypercholesterolemia	3(17.6)	2(8.3)	2(5)
Anemia, n(%)	0(0)	21(87.5)	28(70)
Renal failure, n(%)	0(0)	24(100)	40(100)
Hemodialysis, n(%)	0(0)	24(100)	44(100)
Median hemodialysis duration(range) (mo)	-	81(16–165)	94(12–306)
Laboratory examinations			
Hemoglobin (g/L)	-	106.8	100.9
Product of Ca and Pi (mg^2^/dl^2^)	-	49.4	44.9
iPTH (ng/ml)	-	314.3	210.7
Medication, n(%)			
Aspirin	0(0)	0(0)	1(4.2)
β-blocker	0(0)	8(33.3)	11(27.5)
CCB	0(0)	5(20.8)	18(45)
ACEI	0(0)	3(12.5)	5(12.5)
ARB	0(0)	2(8.3)	4(10)
Statins	0(0)	0(0)	0(0)
Warfarin	0(0)	0(0)	0(0)

PTCA: percutaneous transluminal coronary angioplasty; CABG: coronary artery bypass grafting; β-blocker: β receptor blocker; CCB: calcium-channel blocker; ACEI: angiotensin-converting enzyme inhibitor; ARB: angiotensin II type1 receptor blocker.

### Plasma levels of miR-133a are decreased in MHD patients with LVH compared with those without LVH

**Figure 1:**
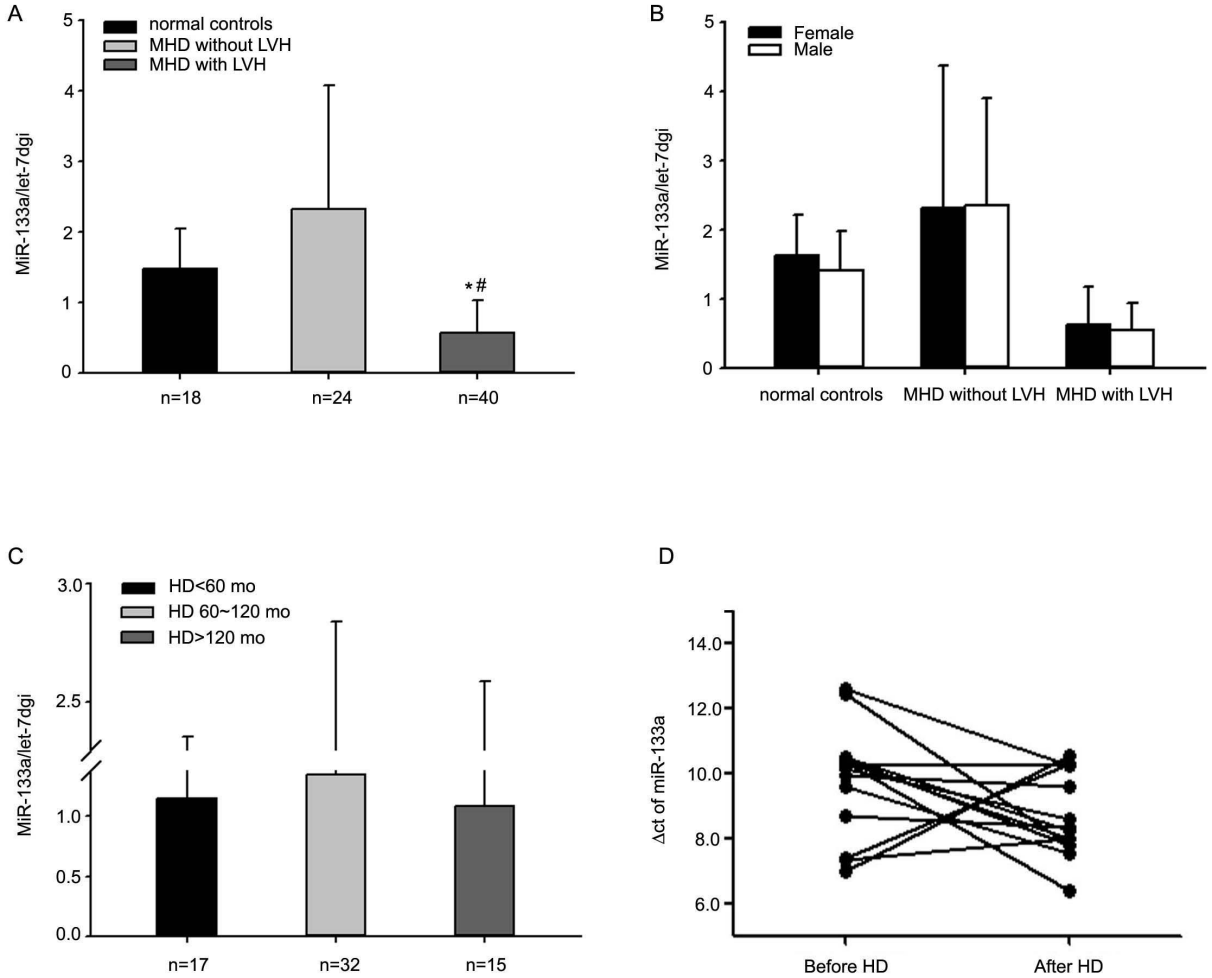
Plasma concentrations of miR-133a in healthy controls and MHD patients. The average miR-133a level of MHD patients with LVH was much lower than that of healthy controls and MHD patients without LVH, (0.57±0.46 *vs* 1.48±0.57, P = 0.000; 0.57±0.46 *vs* 2.33±1.75, P = <0.001) (A). There were no differences in miR-133a level between male and female in controls (1.40±0.58 *vs* 1.63±0.58, P = 0.457) and MHD patients either with LVH (0.54±0.41 *vs* 0.63±0.55, P = 0.565) or without LVH (2.34±1.56 *vs* 2.31±2.07, P = 0.961) (B). When patients were divided into three groups according to the duration of hemodialysis, there was no significant difference in miR-133a levels between groups (1.15±1.21 *vs* 1.39±1.50 *vs* 1.02±1.48, P = 0.806) (C). Among 14 patients receiving hemodialytic treatment, miR-133a concentration remained unchanged after hemodialysis (D). Δct (ct value of 133a subtract ct value of let-7dgi) was used to evaluate the alteration. If Δct was increased, the level of miR-133a was decreased.

To investigate plasma miR-133a levels, blood samples were obtained from healthy controls and MHD patients before hemodialysis. The average level of plasma miR-133a was much lower in MHD plus left ventricular hypertrophy group compared to MHD group, P = <0.001 (Figure 1A). There were no significant differences in miR-133a level between male and female both in healthy controls and MHD patients with or without LVH (Figure 1B). When patients were divided into three groups according to their duration of hemodialysis, there were no significant differences in plasma miR-133a levels between the groups (Figure 1C). The plasma miR-133a levels after hemodialysis were compared to that before hemodialysis in 14 MHD patients and it was found that miR-133a levels were not changed, P = 0.09 (Figure 1D).

### Plasma miR-133a levels are negatively correlated with LVMI and IVS

**Figure 2:**
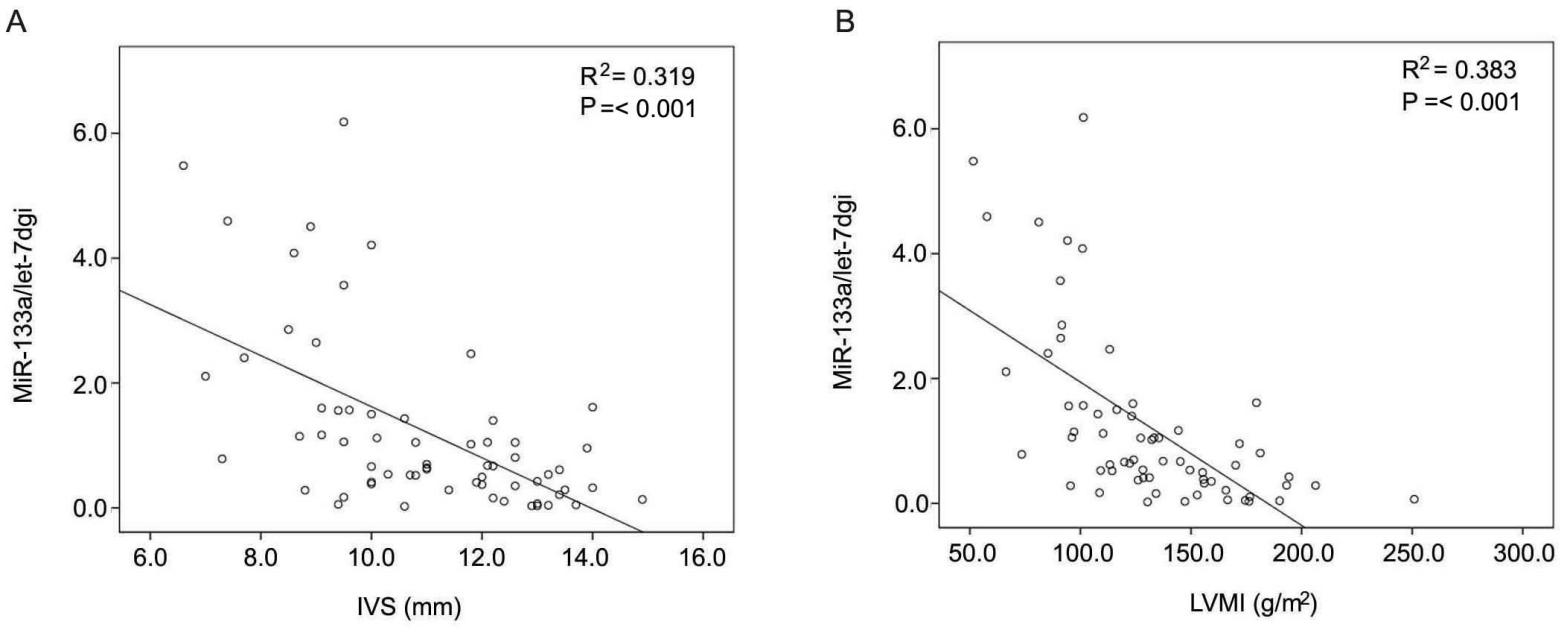
The correlation between plasma miR-133a level and cardiac hypertrophy in ESRD patients receiving maintenance hemodialysis treatment. Liner regression analysis showed that plasma miR-133a concentration was negatively correlated with IVS and LVMI, the correlation coefficient was 0.319 and 0.383 respectively, P = <0.001 (A, B).

Previous study has demonstrated cardiac specific miR-133a control cardiac hypertrophy [17]. Therefore, we estimated the association between plasma miR-133a and cardiac hypertrophy. All of the patients were evaluated with ECHO while 8 of them were measured with MRI. It was confirmed that the findings from echocardiography were consistent with the results of MRI so they were credible (Table 2). To elucidate whether plasma miR-133a concentration were associated with cardiac hypertrophy in MHD patients, the liner regression analysis was used and it indicated that plasma miR-133a level were negatively correlated with IVS and LVMI, R^2^ = 0.319 and 0.383 respectively, P = <0.001 (Figure 2A, B). To further confirm the correlation between plasma miR-133a level and LVH, univariate logistic regression analysis was used to analyze the association of miR-133a level and IVS/LVMI, other clinical characteristics were also analyzed. Table 3 showed that plasma miR-133a level were only negatively correlated with IVS and LVMI, 95% confidence intervals were 0.084 to 0.677 and 0.043 to 0.396 respectively.

**Table 2:** Left ventricular mass index estimated by echocardiography and cardiac magnetic resonance.

patient	echocardiography			cardiac magnetic resonance		
	LVDd	IVS	LVPW(cm)	LVDd	IVS	LVPW(cm)
1	4.45	0.7	0.68	4.0	0.9	0.6
2	4.77	0.86	0.88	5.1	0.8	0.5
3	4.83	0.95	1.03	5.2	0.9	0.5
4	4.07	0.74	0.63	4.1	0.8	0.6
5	4.58	1.29	1.07	4.7	1.1	0.7
6	4.52	1.2	1.05	4.5	1.5	0.7
7	4.66	1.37	1.22	5.0	1.5	0.5
8	5.66	1.32	1.25	5.1	1.4	0.9

**Table 3:** Univariate logistic regression analysis of plasma miR-133a and clinical characteristics.

Variates	95%CI
Gender	0.402–3.043
Age	0.151–1.129
Cardiovascular history	
Hypertension	0.304–7.226
Diabetes	0.074–2.567
Hypercholesterolemia	0.167–22.482
Left ventricular hypertrophy	0.044–0.461*
Anemia	0.406–7.985
Median hemodialysis duration	0.151–1.129
Medication	
β-blocker	0.269–0.306
CCB	0.215–1.686
ACEI	0.313–2.389
ARB	0.074–2.567
Laboratory index	
Troponin I	0.375–5.437
Troponin T	0.229–3.344
LVMI	0.043–0.396*
IVS	0.084–0.677*

CI: confidence interval.

### Plasma levels of cardiac Troponin I and Troponin T were not increased in MHD patients before hemodialysis and not correlated with cardiac hypertrophy

**Figure 3:**
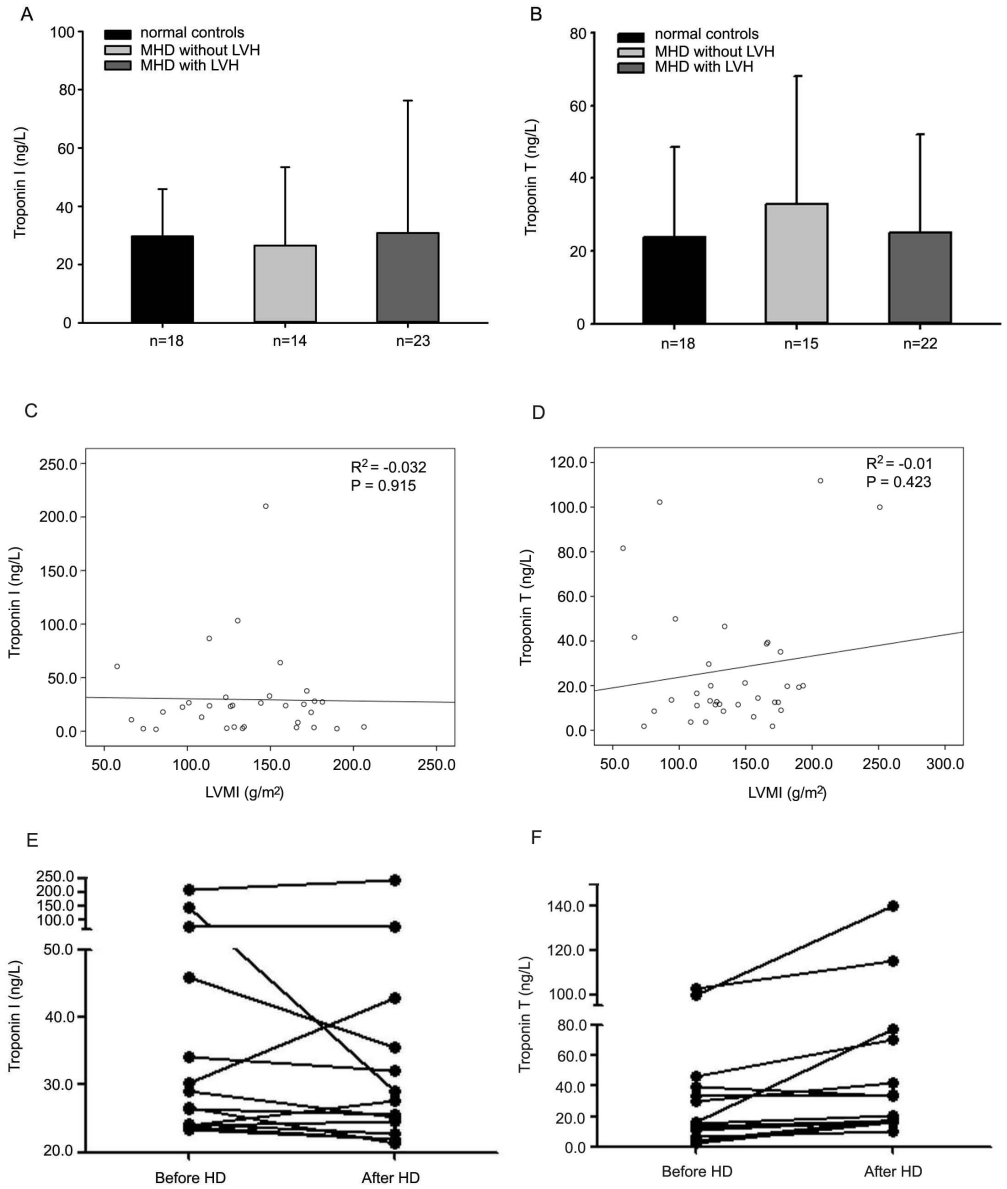
Plasma levels of cardiac Troponin I/T and the correlation between Troponin I/T levels and LVMI/IVS. There were no significant differences between healthy controls and MHD patients in both biomarkers before the hemodialysis treatment (cTnI: 29.72±16.16 *vs* 29.55±40.35, P = 0.983; cTnT: 23.85±24.67 *vs* 27.47±29.16, P = 0.650) (A, B). No significant differences of Troponin I/T levels were found between MHD patients plus LVH and those without LVH (cTnI: 30.85±45.46 *vs* 26.55±26.85, P = 0.784; cTnT: 25.22±26.92 *vs* 33.07±35.07, P = 0.480) (A, B). There were no correlation between LVMI and Troponin I/T before hemodialysis treatment (C, D). After hemodialysis, the cardiac Troponin I level remained unchanged in most patients except that it was evident decrease in one patient (E). There were no significant differences of the plasma Troponin T level between before and after hemodialysis (F).

Cardiac Troponin I and Troponin T are common biomarkers of cardiac infarction. We assessed plasma levels of cardiac Troponin I and Troponin T in MHD patients and healthy controls. Significant differences were not found in both biomarkers between controls and patients (Figure 3A, B). There was no significant correlations between Troponin I/T levels and LVMI (Figure 3C, D). Hemodialysis did not change Troponin I levels except that Troponin I decreased dramatically after hemodialysis in one patient (Figure 3E). Also, there was no significant difference between the Troponin T levels before and after hemodialysis, P = 0.289 (Figure 3F).

## Discussion

In our study, we demonstrated that circulating miR-133a level was decreased in MHD patients compared with healthy controls. Since the expression of miR-133a in heart is decreased in cardiac hypertrophy animal model [17], we analyzed the correlation between circulating miR-133a levels and cardiac hypertrophy in MHD patients. It was found that the miR-133a level was negatively associated with LVMI, an indicator of left ventricular hypertrophy (LVH), in these patients.

The heart has capable of remodeling in response to various environmental demands and a variety of stimuli can induce it to growth or shrink. In MHD patients, primary or secondary hypertension, volume overload, hemodynamic stress and uremia toxins can alone or together induce cardiac hypertrophy.

MicroRNAs are important regulators of a wide range of cellular processes by modulating gene expression and are estimated to regulate more than 30% of the genes in a cell [20]. Recent studies both in animals and humans have demonstrated that miRNAs are present in the circulation and can be detected and quantified [11], [19]. Previous studies have indicated that miR-133a is highly expressed in cardiac and skeletal muscle and is regulated in hypertrophy and failure [17], [21]. Circulating miR-133a levels were measured in several studies and were consistently elevated in patients with cardiac infarction and were closely related with high-sensitivity cardiac Troponin [12], [22], [23], [24].

In the present study, we detected circulating miR-133a levels in MHD patients and healthy controls and demonstrated that miR-133a concentrations were decreased in MHD patients with LVH. Among 64 patients enrolled in our study, most of them (89.4%) had hypertension and about half underwent hypertension more than 5 years, but not all of them had decreased miR-133a levels. The significant association between miR-133a level and LVMI indicated that circulating miR-133a may play important role in the process of LVH and could be a biomarker of LVH. In addition, our study showed that plasma miR-133a concentrations were not changed after hemodialysis. There have been some studies investigated the influence of hemodialysis on circulating microRNA levels [25], [26], [27] and demonstrated different results. Daniel R. and colleagues elucidated miR-499p could be eliminated by hemodialysis [27], however, Thomas Thum [25] and Dai Y [26] indicated that circulating microRNAs could not be cleared by hemodialysis. Although the molecular weight of miRNAs is small enough to permeate the dialysis membrane, it is reported that miRNAs are likely to be transported by larger structures such as proteins and/or microvesicles [28]. Our results implied that miR-133a was not eliminated after hemodialysis. Therefore, we suggest that circulating miR-133a levels before hemodialysis treatment should be used to predict LVH.

Cardiac Troponins are considered the gold standard of biomarkers for the diagnosis of myocardial infarction at present. In our study, no patients suffered from myocardial infarction at the study time. Therefore, the plasma cardiac Troponin-I/T levels were similar to that of healthy controls. Although there was study indicated that cardiac Troponin T concentration positively correlated with left ventricular hypertrophy in hemodialysis patients and predicted lower survival rates [9], we think Troponin-T is more sensitive in cardiac infarction but not hypertrophy.

This study is limited by its small size. In addition, MHD patients differed from controls in terms of age, cardiovascular history, and risk factors. More subjects need to be involved in our future study.

In conclusion, plasma miR-133a levels are decreased in MHD patients with LVH. MiR-133a levels are negatively associated with LVMI, indicating that the lower miR-133a level, the more obvious left ventricular hypertrophy. Our observations supplied the possibility that circulating miR-133a can be a blood-based biomarker for cardiac hypertrophy.
